# A Holistic Approach Towards Evaluating Upper Limb Function in Children with Unilateral Cerebral Palsy: A Narrative Review of Clinical Tools and Promising Technologies for Comprehensive Assessment

**DOI:** 10.3390/jcm14186539

**Published:** 2025-09-17

**Authors:** Giovanna De Luca, Alexandra Kalkantzi, Lisa Mailleux, Rocío Palomo-Carrión, Hilde Feys, Roslyn N. Boyd, Elena Beani, Matteo Cianchetti, Silvia Filogna, Giuseppe Prencipe, Giuseppina Sgandurra, Martina Maselli

**Affiliations:** 1 The BioRobotics Institute, Scuola Superiore Sant’Anna, Viale Rinaldo Piaggio 34, 56025 Pisa, Italy; giovanna.deluca@santannapisa.it (G.D.L.); matteo.cianchetti@santannapisa.it (M.C.); 2 Department of Excellence in Robotics and AI, Scuola Superiore Sant’Anna, Viale Rinaldo Piaggio 34, 56025 Pisa, Italy; 3 Department of Rehabilitation Sciences, KU Leuven, Tervuursevest 101, 3001 Leuven, Belgium; alexandra.kalkantzi@kuleuven.be (A.K.); lisa.mailleux@kuleuven.be (L.M.); hilde.feys@kuleuven.be (H.F.); 4 Child & Youth Institute, KU Leuven, Tiensestraat 102, 3000 Leuven, Belgium; 5 Department of Nursing, Physiotherapy and Occupational Therapy, University of Castilla-La Mancha, Av. de Carlos III, 45004 Toledo, Spain; rocio.palomo@uclm.es; 6 Queensland Cerebral Palsy and Rehabilitation Research Centre, The University of Queensland, Graham Street 62, Brisbane 4101, Australia; r.boyd@uq.edu.au; 7 Department of Clinical and Experimental Medicine, University of Pisa, Via Roma 67, 56126 Pisa, Italy; elena.beani@fsm.unipi.it (E.B.); giuseppina.sgandurra@fsm.unipi.it (G.S.); 8 Department of Developmental Neuroscience, Stella Maris Scientific Institute, Viale del Tirreno, 341, 56128 Pisa, Italy; silvia.filogna@fsm.unipi.it; 9 Department of Computer Science, University of Pisa, Largo Bruno Pontecorvo 3, 56126 Pisa, Italy; giuseppe.prencipe@unipi.it

**Keywords:** cerebral palsy, upper extremity, clinical evaluation, children, wrist-worn sensors, sensorized toys, markerless systems, eye-tracking systems

## Abstract

Optimal upper limb (UpL) function is essential for performing daily activities; however, children with unilateral spastic cerebral palsy (USCP) often experience impairments in UpL function, which can impact their quality of life or independence. While UpL motor impairments are a primary concern, non-motor functions, such as cognition, attention, and visual function, commonly impaired in USCP, may also play a role in UpL performance. Nevertheless, these non-motor functions are often not considered in evaluation protocols that focus on the UpL. Moreover, clinical evaluation is typically conducted in structured and controlled settings and may not accurately reflect the child’s abilities in daily life. Non-invasive, novel technologies are a promising solution for filling this gap, by providing additional quantitative and ecologically valid information to clinicians. In this context, this overview aims (i) to present the most frequently used tools for a holistic evaluation in children with USCP, ensuring a thorough understanding of the UpL function, and (ii) to report the evidence of how novel, non-invasive technologies can enhance clinical evaluation in daily life, enabling a more comprehensive evaluation. This work could set a basis for multidimensional evaluation protocols for UpL function in USCP, providing a different approach to the current standards.

## 1. Introduction

Cerebral palsy (CP) refers to a non-progressive disorder attributed to a lesion in the developing brain, which affects about 18 million individuals worldwide. The CP prevalence accounts for 1-2/1000 live births in high-income countries, making it one of the most common causes of childhood-onset disability [1]. As a direct consequence of the brain lesion, children with CP experience movement and posture abnormalities leading to functional impairments. One of the most common CP subtypes—accounting for more than 1/3 of the total population with CP—is predominantly spastic unilateral CP (USCP), where the lesion affects mainly one side of the body [2]. Children diagnosed with USCP usually achieve independent walking, yet with restrictions in walking speed or quality [3]. Nevertheless, the impairments in the upper limb (UpL) are more prominent and are considered widely responsible for daily challenges, such as difficulties with self-care and independence [4]. Overall, the presence of motor impairments reduces the children‘s ability to learn and adapt mainly bimanual actions into their daily life, including play, self-care, and academic activities [5].

Despite sharing the same diagnosis, children with USCP usually exhibit large variability in the clinical presentation of the UpL, in terms of muscle strength and muscle tone, range of motion, and somatosensation, but also in terms of manual ability, dexterity, unimanual capacity, and bimanual performance [6,7]. Studies in USCP have shown that there is also variability in UpL functional outcomes, as non-motor functions differ in this population and can also determine the level of functionality [8,9]. For example, apart from disruption in the descending motor pathways, impairments in UpL function have also been associated with disruption in the ascending thalamocortical pathways/somatosensory cortical areas, and the visual and the pre(frontal) cortex [10,11,12], compromising sensorimotor and visuomotor integration, motor learning, and anticipatory motor planning [13,14,15]. Moreover, intellectual disability or difficulties with complex cognitive functions can further affect the motor development of the UpL, by impeding the ability to execute fine motor tasks, which are considered more complex [12,16]. This suggests that a single-dimensional evaluation, addressing only motor aspects, might overlook important aspects of UpL function. Therefore, UpL function should be assessed through a multidimensional evaluation, essential to map functional abilities, identify areas of attention, monitor progress over time, and set relevant therapeutic goals, tailored to each child’s needs.

Over the last few decades, various clinical performance-based tests, questionnaires, and classification systems have been developed to serve as a structured way of assessing the functional abilities of individuals with CP. The development of clinical tools always occurs in concert with the International Classification of Functioning, Disability, and Health in Child and Youth (ICF-CY), the universal framework developed by the World Health Organization [17] to describe and assess health, functioning, and disability from a holistic perspective. Particular attention has been given to bias elimination, and the consistency and reproducibility of the results stemming from clinical tools, with studies establishing population-specific psychometric properties.

While clinical assessments have increased clinical and scientific value and are being routinely used in the clinical and research setting, they often lack objective quantitative data, as they are mediated by the evaluator’s experience [18]. Furthermore, conducting clinical assessments within controlled environments (i.e., clinical or research settings) may not provide a representative example of the child’s behavior in daily life [19]. As Van der Lee et al. highlighted, “the best way to obtain valid information about the use of the affected arm and hand in daily life would obviously be to observe the patient in the home environment” [20]. In the state of the art, numerous studies on technologies have been conducted to help clinicians in obtaining objective data from the assessment of the UpL, while maintaining ecological validity. Intending to create assessments that are unobtrusive, there is increasing attention towards technologies that are minimally invasive and can be used in daily life without the strict presence of a clinician.

Several reviews have already advanced knowledge in this field, each focusing on specific aspects of UpL evaluation in children with CP or motor impairments. For instance, systematic reviews have summarized instrumented assessments that evaluate UpLs [21] and bimanual movements in CP [22], clinical tools for unimanual skills in CP [23], and mobile health technologies for the assessment of and intervention for UpLs in children with motor impairments [24]. However, none of these studies have synthesized tools that assess factors beyond motor impairments to provide a holistic view of UpL abilities. Moreover, a comprehensive overview of emerging non-invasive technologies that can support the understanding of UpL function in daily life is still lacking. This study therefore aims to bridge this gap by presenting a holistic approach to evaluating UpL function in children with USCP, integrating both established clinical tools and promising technologies for a more comprehensive assessment. The use of technological solutions in the clinical context can have a complementary role by capturing spontaneous performance during daily activities, which is often difficult to assess through standardized clinical tests alone.

Based on the number of various tools available for the evaluation of children with CP, we focused on the following research aims: (i) to present the most frequently used clinical assessments to evaluate the UpL function of children with USCP, within the frame of a holistic approach; (ii) to report the evidence of how non-invasive promising technologies can enhance clinical UpL evaluation in children with USCP in daily life, enabling a more comprehensive evaluation. This review is structured to first describe the literature search, followed by an overview of clinical assessments and promising technological approaches. Next, a summary of the findings is presented, including the advantages and limitations of each approach. We then discuss how promising technologies can complement clinical assessment, and highlight implications for practice, before concluding with recommendations for future research. It is aspired that this work will set the foundation for a unified framework for assessing UpLs in children with USCP, supporting more comprehensive and ecologically valid evaluations.

## 2. Methods

This study is a non-systematic narrative literature review. Literature was identified through searches in four international databases relevant to the clinical field (PubMed, MEDLINE, CINAHL, and Embase) as well as in two more technologically oriented databases (Web of Science and Scopus), using combinations of keywords and Boolean operators to capture variations in terminology. Where available, systematic reviews were also included to guide recommendations for the selection of the assessments. Studies were considered if they involved children with CP, especially USCP, and used assessment tools mapped on the ICF-CY framework that had established psychometric properties according to COSMIN standards [25]. The documented clinical assessments (e.g., classification systems, performance-based tests, questionnaires)—chosen for their prominence in the literature—are the most frequently used in children with USCP and primarily target those over the age of five, aligning with the age range supported by most standardized evaluation tools. For the technologies, we also considered those not yet applied to children with USCP, provided that there is sufficient evidence to support their potential. In addition, the selected technologies were characterized by low invasiveness and high portability, as they do not rely on wearable sensors or a structured environment. However, wrist-worn sensors were also considered, as they are commonly accepted. The end selection was based on relevance and a consensus among the authors, reflecting their collective experience with clinical tools, technologies, and familiarity with commonly used tools in both research and practice.

## 3. Overview of Clinical Assessments

The overview of the clinical assessments (Table 1) will be synthesized according to the different levels of the ICF-CY [17] to ensure comprehensive coverage of all areas of functioning in children with USCP. The ICF-CY levels into which the clinical assessments will be divided are Body Function and Structure, Activity, Participation, and Quality of Life.

### 3.1. Body Function and Structure Level

#### 3.1.1. Motor

Motor impairments mainly encompass increased muscle tone (most often spasticity), muscle weakness, and decreased muscle length. The increased muscle tone is mainly present in the antigravity muscles, with distal muscles being more involved compared to proximal muscles [4]. Essentially, this results in an UpL flexion pattern, with flexed fingers, wrist, and elbow; a pronated forearm; and a protracted and endorotated shoulder. Increased spasticity may further result in fisting and difficulties with opening the hand and extending/abducting the fingers. Muscle weakness is also more prominent distally [26]. Overall, the affected hand can be described as slow and weak, with uncoordinated movements and spasticity, accompanied by difficulties in grasping, releasing, and manipulating objects [27]. Klingels et al. stated that these types of motor impairments can be assessed reliably in children with USCP over the age of 5 years [26].

The passive range of motion (ROM) is traditionally evaluated with a universal ***goniometer***, a device that measures the angle of a joint or permits the rotation of an object to a definite position. The resulting value per joint is interpreted against established normative values. Muscle tone is widely assessed in clinical practice and research with the ***Modified Ashworth Scale (MAS)*** in children with USCP [26]. The MAS allows assessing muscle spasticity with the following grading [28]: 0: no increase in muscle tone; 1: slight increase in muscle tone, with a catch and release or minimal resistance at the end of the range of motion when an affected part(s) is moved in flexion or extension; 1+: slight increase in muscle tone, manifested as a catch, followed by minimal resistance through the remainder (less than half) of the range of motion; 2: a marked increase in muscle tone throughout most of the range of motion, but affected part(s) are still easily moved; 3: considerable increase in muscle tone, passive movement difficult; and 4: affected part(s) rigid in flexion or extension. Muscle strength is widely evaluated using manual muscle testing and scored with the ***Medical Research Council (MRC)*** scale [29]. The patient is instructed to perform an active muscle movement, activating the target muscles, and a score is given, corresponding to the level of strength: 0: no muscle contraction; 1: slight contraction, but no movement; 2: movement occurrence, but no antigravity movement; 3: antigravity movement, but no resistance; 4: movement against moderate resistance; 5: movement against full resistance. Furthermore, based on the systematic review of Dekkers et al. [30], the ***Jamar Dynamometer*** is recommended to be used in CP to evaluate grip strength, defined as the isometric muscle strength of the hand and forearm during grasp. Three trials are performed for each hand, starting with the less-affected hand, and the mean value for each hand is recorded.

Another important motor aspect, a direct consequence of the brain injury, is the presence of mirror movements, defined as the involuntary movement of the contralateral hand when the ipsilateral hand moves voluntarily. An easy way to assess mirror movements is the ***Woods and Teuber scale*** [31]. Three repetitive unimanual tasks are performed with each hand: fist opening and clenching, sequential finger opposition, and alternate finger tapping. An alternative test set consists of the following tasks: fist opening and clenching, repetitive index tapping to the thumb, and simultaneous finger tapping on the table. The tasks are videorecorded and scored accordingly: 0: no clearly imitative movement; 1: barely discernible repetitive movement; 2: either slight but unsustained repetitive movement or stronger, but briefer, repetitive movement; 3: strong and sustained repetitive movement; 4: movement equal to that expected for the intended hand [31].

#### 3.1.2. Somatosensory Function

Somatosensory impairments may further complicate motor activity. For example, disturbed somatosensory input from the affected hand may lead to reduced internal representation of the object properties, producing a reduction in the ability for optimal object manipulation or effective grasp. This interplay between somatosensory function, manual ability, and processing skills leads to changes in anticipatory control, influencing functional outcomes [32]. Up to 90% of the children with CP have somatosensory impairments, including either difficulties with registering tactile input and/or difficulties with interpreting somatosensory input (i.e., tactile perception) [13,33].

A traditional assessment of somatosensory function includes the evaluation of proprioception, tactile registration, and tactile perception. Proprioception is usually assessed through the passive movement sense evaluation of the index. With the vision occluded, the index is passively moved by the therapist within, initially, small ranges, while expecting an indication by the patient when the movement is felt. Tactile registration is usually evaluated with ***Semmes****–**Weinstein monofilaments***. First, a 0.07 g monofilament is applied to the index fingertip, expressing normal tactile registration. Based on the patient’s performance, a lighter (more difficult) or heavier (easier) monofilament is used afterwards [34]. Tactile perception can be evaluated with the assessment of stereognosis and two-point discrimination. To assess ***stereognosis***, ***12daily-life objects*** (i.e., six paired—pen and pencil, coin and button, and paper clip and safety pin—and six unpaired: tennis ball, spoon, comb, clothespin, marble, and key) are given to the patient with their vision occluded and they are asked to identify the objects by only touching them. Two-point discrimination, defined as the ability to differentiate that two nearby objects touching the skin are truly distinct points, is routinely assessed with an ***Aesthesiometer***. The two prongs of the Aesthesiometer initially have a 4 mm distance from each other, which is progressively decreased. The smallest distance at which the patient feels two distinct points is recorded [26].

#### 3.1.3. Visual Function

Cerebral visual impairment (CVI), defined as a ‘verifiable visual dysfunction not attributed to disorders of the anterior visual pathways or any potentially co-occurring ocular impairment’, occurs in up to 70% of children with CP [35], with the visual-specific problems varying based on the CP subtype. Particularly for children with USCP, impairments in visual function usually appear in terms of reduced visual acuity, hemianopia, disturbed stereoacuity, and impaired visual–perceptual function, affecting up to 62% of the population [35,36,37]. In CVI, the processing of visual information is disturbed, potentially impacting the motor planning and finetuning of UpL movements, especially those requiring both hands. Hence, the evaluation of visual–perceptual skills is essential, within the context of acquiring a comprehensive insight into the UpL abilities.

A test widely used in children with USCP is the ***Test of Visual Perceptual Skills—Fourth version (TVPS-4)***, a non-motor test which comprehensively assesses seven dimensions of visual perception i.e., visual discrimination, visual memory, spatial relationship, form constancy, sequential memory, visual figure-ground, and visual closure [38]. Similar conclusions apply for the ***Developmental Test of Visual Perception—Third edition (DTVP)***, which evaluates eye–hand coordination, copying, figure-ground, visual closure, and form constancy, while exhibiting acceptable internal consistency and convergent validity with the Beery-VisuoMotor Integration test and the ***Motor-Free Visual Perceptual Test (MVPT)***, evaluating visual discrimination, visual figure-ground, visual memory, visual closure, and visuospatial aspects [39].

#### 3.1.4. Cognitive Function

Most children with USCP exhibit typical levels of cognition, with about 19% having an intellectual quotient (IQ) level below 70, which can further compromise their level of functionality [40]. However, the difficulties encountered by children with USCP do not appear to be limited to general cognitive functioning but also include behavioral and emotional control processes. Specifically, about 1/3 of the population with USCP experience specific learning impairments or problems in executive functions (EFs) [41,42,43]. EFs refer to a set of cognitive processes involved in novel situations, and in complex and goal-directed actions and allow individuals to regulate their own cognitive processes and behaviors [44]. The three core EFs are inhibition (i.e., the ability to suppress inappropriate responses and inhibit distractors); working memory (i.e., the ability to keep information in mind while performing mental operations on it); and shifting or cognitive flexibility (i.e., the ability to flexibly shift between ideas and activities [44]).

Starting from general intellectual functioning, the tool traditionally used for its assessment is the ***Wechsler Preschool and Primary Scale of Intelligence—Fourth Edition*** (***WPPSI-IV***) for children aged 2.64–7.7 years [45]. The WPPSI-IV provides measurements of the Full-Scale IQ (FSIQ) and the following five primary indices: the Verbal Comprehension Index (VCI), Visual Spatial Index (VSI), Fluid Reasoning Index (FRI), Working Memory Index (WMI), and Processing Speed Index (PSI). Ancillary indices can also be calculated for the Vocabulary Acquisition Index (VAI), Nonverbal Index (NVI), General Ability Index (GAI), and Cognitive Proficiency Index (CPI). Moreover, to reliably assess intellectual functioning in older children, the ***Wechsler Intelligence Scale for Children—Fifth Edition (WISC-V)***, for children ages 6–16 years, is used. The WISC-V [46] is a test of general intelligence composed of 16 subtests expressed as scaled scores. There are seven primary subtests that determine the FSIQ, used to produce the five-factor index scores (two subtests each for the Verbal Comprehension Index [VCI], Visual Spatial Index [VSI], Fluid Reasoning Index [FRI], Working Memory Index [WMI], and Processing Speed Index [PSI]) [47].

EFs in daily life are traditionally assessed with the ***Behavior Rating Inventory of Executive Functioning (BRIEF)***, a questionnaire developed in self-reported, parent-reported, and teacher-reported form [48]. The items of the BRIEF describe daily situations in which behavioral expressions of EFs can be exhibited. Furthermore, various performance-based assessments are being used to evaluate EFs in CP, according to the systematic review of Pereira et al. and other studies investigating EFs in USCP [49]. For example, inhibition can be assessed with the ***Conners continuous performance test*** [50], which evaluates four dimensions of attention in a computerized format. A series of visual stimuli is presented, one-at-a-time, to the participant, who is instructed to respond to target stimuli while inhibiting their response to non-target stimuli. Another example is the ***Stroop test*** or the ***Color–Word Interference Test (Inhibition Condition) of the D-KEFS***, in which the participant names the ink color of a color word that is incongruent with the word itself. In addition, working memory is frequently assessed with the ***Digit Span Backward subtest of the WISC-IV***, where the participant hears a sequence of digits and is asked to repeat them in reverse order, or the ***Corsi block-tapping test***, where the participant is asked to retrieve a sequence previously seen performed by the examiner by tapping blocks with the preferred finger, following the same order for the forward condition or reversing the order for the backward condition. Lastly, shifting or cognitive flexibility can be evaluated using the ***Trail Making Test***, where individuals should connect a sequence of numbers and then alternate between numbers and letters in order, or the ***Verbal Fluency Test from the D-KEFS***, for which individuals are requested to generate as many words as possible under specific constraints within a time limit [49]. However, it should be noted that a common challenge when assessing EFs is the so-called ‘task impurity’. This phenomenon manifests because some tasks assessing one EF (e.g., working memory) might also assess another EF (e.g., shifting), due to the overlap in characteristics and the close interrelation between the different EFs. To minimize task impurity, the literature suggests the use of multiple measures to assess each distinct EF [51].

### 3.2. Activity Level

Activity is defined as one’s capacity to perform a task or action. Based on the scoping review of Chagas et al. [52], the ICF-CY Activity level is the most investigated in CP, with most studies focusing on mobility, carrying, moving, and handling objects. To evaluate the activity of the UpL, a number of classification systems, performance-based assessments, and questionnaires have been developed or adapted for children with CP.

Starting with the classification systems, the ***Manual Ability Classification System (MACS)*** is traditionally used. The MACS classifies the child’s ability to handle objects in daily activities at one out of five levels (level I: greater ability; level V: less ability) and is applicable in children from 4 to 18 years old. Typically, in USCP, children are mostly classified in MACS levels I to III: MACS level I (able to handle objects easily and successfully), level II (able to handle most objects but with somewhat reduced quality and/or speed of achievement so that alternate ways of performance might be used), or level III (handles objects with difficulty; needs help to prepare and/or modify activities) [53]. Moreover, the ***Bimanual Fine Motor Function—Version 2 (BFMF2)*** is used to classify bimanual fine motor function in children with CP aged between 3 and 18 years. BFMF2 is also based on a five-level scale (level I: greater function; level V: the most limited function [54]). The BFMF2 offers the possibility to classify the capacity of the two hands separately, in contrast to MACS, which classifies the performance of both hands together. The classification levels could be determined from three fine motor tasks, regardless of bimanual fine motor ability, making the BFMF2 a useful tool for classifying fine motor capacity. Lastly, the ***House Functional Classification System (HFCS)*** assesses the UpL function of the affected hand. It comprises a nine-point scale that categorizes hand function, ranging from no use of the hand to use as a passive assist, an active assist, and ultimately to *spontaneous use of the affected hand* during tasks [55,56].

With respect to clinical performance-based assessments, the ***Assisting Hand Assessment (AHA)*** has been widely used in children with USCP and is defined as the *gold standard* for evaluating bimanual performance. Essentially, the AHA assesses the spontaneous use of the affected hand in bimanual tasks, and it comprises a semi-structured play session, involving objects that trigger bimanual activity [57]. The session is recorded, and the video is scored according to 20 aspects. Furthermore, ***the Melbourne Assessment of Unilateral Upper Limb Function (MUUL)*** [58], with its current updated version ***Melbourne Assessment 2 (MA2)***, is a reliable and valid assessment for children with neurological impairment. The MA2 consists of 14 unimanually tested items including reaching, pointing, manipulating, grasping, releasing, and pro-/supination. Standardized instructions describe how to administer and videotape the assessment. Each item is scored from the videotape on a three-, four-, or five-point ordinal scale, with the scoring divided into four subscales: range of motion, accuracy, dexterity, and fluency [59]. The ***Box and Blocks Test (BBT)*** is a reliable and valid assessment for children with USCP [60], designed to measure manual dexterity. Specifically, it evaluates how quickly and efficiently an individual can transfer small wooden blocks from one compartment of a box to another, within 60 s. The BBT is a standardized assessment that is portable, readily accessible, easy to use, and quick to administer [61].

Lastly, questionnaires can provide additional information about the child’s functional level. The ***ABILHAND-kids questionnaire*** [62] is a Rasch-based measure of manual ability used in children with CP. It captures the ability of children to perform activities of daily living, independent of the strategies involved. [63] Due to its parent-reported nature, it relies on parental recall within the preceding three months, with each item scored on a three-point scale (i.e., impossible, difficult, easy). The ***Children’s Hand-use Experience Questionnaire (CHEQ)*** was developed based on the dual premise that the efficient completion of daily-life activities requires the skillful use of both hands together and that a person’s perception of competency contributes to a comprehensive understanding of the UpL function. The CHEQ was designed for children and adolescents with USCP, UpL reduction deficiency, or obstetric brachial plexus palsy. Its development aimed to capture the individual’s experience while using the affected hand in daily activities that typically require both hands. Lastly, the ***Caregiver Functional Use Survey (CFUS)*** was designed to assess caregivers’ perceptions on how often and how well children with USCP use their affected UpL in ‘real-life’ situations, on a six-point scale [64].

### 3.3. Participation Level

Participation refers to one’s involvement in life situations and the ability to engage in societal roles. For children with USCP, participation across settings might be limited due to the burden imposed mainly by their physical abilities. An essential aim of rehabilitation is to improve functional ability and consequently enhance participation in meaningful activities for people with living experience [65]. This implies that goal-setting must occur in a patient-centered manner, based on the priorities of the child and their family.

In the clinical reality, a few assessment tools are being used to either assess the level of participation or assist in goal-setting. Based on the systematic review of James et al. [66], the ***Assessment of Motor Processing Skills (AMPS)*** is the best measure for evaluating performance in daily life [67]. Additionally, the systematic review of Sakzewski et al. suggested the use of the ***Canadian Occupational Performance Measure (COPM)*** to evaluate participation outcomes in children with CP. The COPM is a personalized goal-setting tool and outcome measure that facilitates the identification of meaningful patient-centered goals related to daily activities and has additionally shown adequate responsiveness in detecting clinically significant changes. Lastly, Sakzewski et al. also recommended the use of the ***Participation and Environmental Measure for Children and Youth (PEM-CY)*** [68] as an adjunct within an evaluation protocol, to capture participation at home, at school, and in community environments, based on parental views.

### 3.4. Quality of Life Level

Quality of Life is a broad concept encompassing various components of overall health and well-being (e.g., physical, psychosocial, economic, and cultural). It can be influenced by the context of the culture and value systems and relates to the individual’s goals, expectations, and concerns [69]. The life quality of children and adolescents with a neurological condition can be impacted at various levels, including in physical (e.g., physical health, independence in basic functional activities), psychological (e.g., mental status, positive self-perception), and psychosocial dimensions (e.g., forming friendships, leisure time, finding a partner). Identifying the factors that are associated with better or worse life quality is important to guide therapeutic strategies and the allocation of resources, thus contributing to optimizing the well-being of these children, youth, and young adults [70]. In the clinical and research setting, life quality is mainly assessed with self-reported, qualitative measures (i.e., questionnaires), such as the ***Child Health Questionnaire (CHQ)***; ***Youth Quality of Life Instrument—Research version (YQoL-R)***; (excluding subscales related to service access and pain/feeling about disability); ***Pediatric Quality of Life Inventory (PedsQL)****; **Children’s Quality of Life (TACQOL)***; and ***Schedule for the Evaluation of Individual Quality of Life (SEIQoL)*** [71,72]. One of the most used life quality measures in CP, also widely applicable in USCP, is the ***Cerebral Palsy Quality of Life Questionnaire (CP-QoL)***, as it is a condition-specific measure.

Table 1 provides a summary of the classifications and clinical tools discussed, organized by the ICF-CY domains.

**Table 1:** Validated or most frequently used clinical assessments and classifications in children with unilateral spastic cerebral palsy.

ICF-CY	Metrics	Clinical Tools	Psychometric Properties	Condition	Age Range
**Body Function and Structure**	Range of motion (=the extent to which a joint can move within its physiological plane of movement)	Universal goniometer [26]	-Inter-Rater Reliability for Wrist and Elbow: ICC = 0.48–0.73 [26]. -Test–Retest Reliability: ICC = 0.81–0.94 [26].	USCP	≥5 years
	Muscle tone (=the muscle’s resistance to passive stretch during resting state)	MAS [26]	-Inter-Rater Reliability: ICC = 0.64–0.88 [26]. -Test–Retest Reliability: ICC = 0.57–0.90 [26].	USCP	≥5 years
	Muscle strength (=the ability of a muscle or group of muscles to exert force against resistance)	MRC scale [29]	-Inter-Rater Reliability for Individual UpL Muscle Groups: ICC = 0.49–0.90 [26]. -Test–Retest Reliability: ICC = 0.69–0.96 [26].	USCP	≥5 years
	Grip strength (=specific measure of the force generated by the muscles of the hand and forearm)	Jamar dynamometer [26]	-Inter-Rater Reliability: ICC = 0.95 [26]. -Test–Retest Reliability: ICC = 0.96 [26].	USCP	≥5 years
	Mirror movements (=involuntary movements on one side of the body that mirror voluntary movements occurring on the opposite side)	Woods and Teuber scale [31]	-Inter-Rater Reliability: ICC = 0.90. -Intra-Rater Reliability: ICC = 0.92 [31].	USCP	≥5 years
	Tactile registration (=the ability to detect and respond to touch stimuli on the skin)	Semmes–Weinstein monofilaments	-Reliability in the Affected Hand: ICC = 0.96 [73]. -Reliability in the Unaffected Hand: ICC = 0.90 [73]. -Convergent Validity in the Index Finger: ρ = 0.57–0.65. -Inter-Rater Reliability in the Index Finger: kw = 0.79. -Intra-Rater Reliability in the Index Finger: kw = 0.80 [74].	USCP TD	5–17 years
	Stereognosis (=the ability to recognize and identify objects only by touch)	12 daily-life objects [26]	-Inter-Rater Reliability: ICC = 0.78 [26]. -Test–Rest Reliability: ICC = 0.86 [26].	USCP	≥5 years
	Two-point discrimination (=the ability to determine the smallest distance at which two separate touch points can be perceived as distinct)	Aesthesiometer [26]	-Inter-Rater Reliability: ICC = 0.92 [26]. -Test–Retest Reliability: ICC = 0.81 [26].	USCP	≥5 years
	Visual-perceptual function (=the ability to interpret, analyze, and give meaning to visual information received from the environment)	TVPS-4; DTVP; MVPT [39]	-TVPS-4 Internal Consistency: Cronbach’s a = 0.94. - DTVP Internal Consistency: Cronbach’s Coefficients ¼ 0.8–0.97 for subtests. 0.93 or above for composites. -MVPT Internal Consistency Cronbach’s a: 4–10 years, 0.69–0.87. 4–11 years, 0.86–0.9. -TVPS Test–Retest Reliability: Whole Test ICC = 0.88; Subtests ICC 0.38–0.77. -DTVP Test–Retest Reliability: r = 0.95. -MVPT Test–Retest Reliability: 4–10 years r = 0.87; >11 years r = 0.92 [39].	Hemiplegia	≥4 years
	Cognitive function (=the overall capacity of an individual to think, reason, learn, and adapt)	WPPSI-IV; WISC-V [41]	-WPPSI-IV Internal Consistency for the Composite Scales: ICC = 0.86 to ≥0.90 -WPPSI-IV Test–Rest Reliability: ICC = 0.84–0.93. -WPPSI-IV Inter-Rater Reliability: ICC = 0.96–0.99 [75]. -WISC-V Criterion Validity: ICC = 0.89. -WISC-V Test–Retest Reliability: ICC = 0.86–0.93 [76].	TD	2.64–7.7 years and 6–16 years
	Executive functions (=a set of advanced cognitive processes enabling goal-directed behavior, planning, decision-making, problem-solving, and self-regulation)	BRIEF; Conners continuous performance test (cpt); Stroop test; digit span; Corsi block-tapping test; trail-making; tests of verbal fluency [49]	-BRIEF: Good stability over time [77]. -Conners cpt (Internal, Consistency): Cronbach’s a= 0.64–0.96. -Conners cpt Test–Retest Reliability: ICC = 0.48–0.79 [50]. -Corsi Block-Tapping Test: Good concurrent validity with the WISC [78].	USCP TD TD	8–17 years; mean age: 20.01 years >5 years
**Activity**	Functional use of hands/classification (=effective and purposeful use of one or both hands to perform everyday activities)	MACS; BFMF; HFCS	-MACS Inter-Rater Reliability: ICC = 0.97 [53] -BFMF Construct Validity with the MACS: ρ = 0.89 [54].	CP	4–18 years 3–18 years
	Bimanual performance (=the spontaneous use of the affected hand during bimanual tasks)	AHA [79]	-Internal Scale Validity: ICC = 0.98 [80]. -Inter-Rater Reliability: ICC = 0.97 [81]. -Test–Retest Reliability: ICC = 0.99 [81].	USCP	18 months –18 years
	Upper limb movement quality (=the characteristics defining how a movement is performed, focusing on its range of movement, accuracy, dexterity and fluency)	MUUL; MA2 [58]	-MUUL Internal Consistency: PSI up to 0.92. -MUUL Responsiveness to Change: Questionable [58]. -MA2 Test–Retest Reliability: ICC= 0.92–0.98 [82].	CP	2.5–15 years
	Manual dexterity (=the skillful and coordinated use of the hands and fingers to perform precise and complex movements)	BBT [60]	-Responsiveness To Change: More affected hand, effect size = 0.678; Less affected hand, effect size = 0.514 [60].	USCP	5–17 years
	Manual ability (=ability to effectively use both hands to perform everyday activities that require fine skills)	ABILHAND-Kids [62]	-Test–Rest Reliability: ICC = 0.92 [62].	USCP	6–17 years
	Personal perception on upper limb function	CHEQ; CFUS [64,83]	-CHEQ Validity: Effective operational range >90% [84]. -CHEQ Test–Retest Reliability: ICC = 0.88–0.91 [84].	USCP	CHEQ: 3–18 years CFUS: 5–15 years
**Participation**	Goal-setting, assessment of participation	AMPS; COPM; PEM-CY [67,85,86]	-AMPS Test–Retest Reliability: ICC = 0.86–0.93. -AMPS Intra-Rater Reliability: ICC = 0.96–0.98 [66]. -COPM Test–Retest Reliability: ICC = 0.88–0.89 [87]. -PEM-CY Internal Consistency: Cronbach’s a = 0.59–09.1. -PEM-CY Test–Retest Reliability: ICC = 0.58–0.95 [88].	CP TD	5–18 years 5–13 years 5–17 years
**Quality of Life**	Life Quality	CP-QoL; CHQ; YQoL-R; PedsQL; TACQOL; SEIQoL [89]	-CP-QoL Internal Consistency: Cronbach’s a = 0.74–0.92 for primary caregivers. Cronbach’s a = 0.80–0.90 for child self-report. -CP-QoL Test–Retest Reliability for caregiver: ICC = 0.76–0.89. -CP-QoL Concurrent Validity with CHQ, KIDSCREEN, and GMFCS [90,91].	CP	≥4 years

ICF-CY = Functioning and Disability in Child and Youth; USCP = unilateral spastic cerebral palsy; MAS = Modified Ashworth Scale; MRC = Medical Research Council; UpL = upper limb; TVPS-4 = Test of Visual Perceptual Skills-Fourth version; DTVP = Developmental Test of Visual Perception—Third Edition; MVPT = Motor-Free Visual Perceptual Test; WPPSI-IV = Wechsler Preschool and Primary Scale of Intelligence—Fourth Edition; WISC-V = Wechsler Intelligence Scale for Children—Fifth Edition; BRIEF = Behavior Rating Inventory of Executive Function—Second Version; MACS = Manual Ability Classification System; BFMF = Bimanual Fine Motor Function; HFCS = House Functional Classification System; AHA = Assisting Hand Assessment; CHEQ = Child hand-use Experience Questionnaire; MUUL = Melbourne Assessment of Unilateral Upper Limb Function; MA2 = Melbourne Assessment 2; BBT = Box and Blocks test; CFUS = Caregiver Functional Use Survey; AMPS = Assessment of Motor Processing Skills; COPM = Canadian Occupational Performance Measure; PEM-CY = Participation and Environmental Measure for Children and Youth; CP = cerebral palsy; TD = typically developing children; ICC = Intraclass Correlation Coefficient; CP-QoL = Cerebral Palsy Quality of Life; CHQ = Child Health Questionnaire; YQoL-R = Youth Quality of Life Instrument—Research Version; PedsQL = Pediatric Quality of Life Inventory; TACQOL = Children’s Quality of Life; SEIQoL= Schedule for the Evaluation of Individual Quality of Life. Underlined items: questionnaires.

## 4. Overview of Technologies

**Figure 1:**
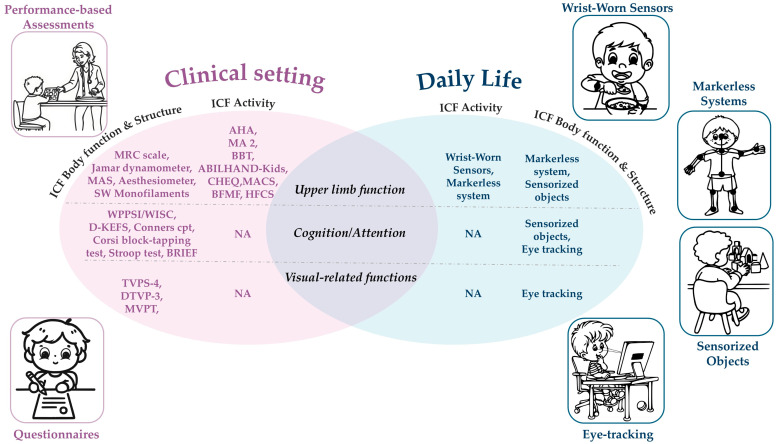
Clinical tools and promising technologies for a comprehensive evaluation of the UpL in children with USCP across the ICF-CY Body Function and Structure and Activity levels.

This section will present wrist-worn sensors, markerless systems, sensorized objects, and eye tracking (Table 2) as promising technologies that best align with our criteria of ecological validity, minimal invasiveness, and applicability in daily life. These technologies could provide a more comprehensive clinical evaluation, targeting the ICF-CY (Figure 1).

### 4.1. Wrist-Worn Sensors

Over the years, wearable sensors have been significantly advanced, enabling the objective and long-term monitoring of motor activities in daily life. These technologies provide an opportunity to improve clinical assessments by capturing real-world data that is often missing from controlled environments. In particular, the triaxial accelerometer is the most used, and it seems to be the most suitable and reliable for monitoring and collecting consistent data about UpL movement [92].

Wrist-worn sensors have emerged as a popular and practical solution for monitoring daily activities. Their design allows individuals to move freely without restrictions, making them particularly applicable for assessing children with USCP, enabling a deeper understanding of UpL function in daily life as well as task-specific abilities. Several wristband devices are commercially available, such as ActiGraph (ActiGraph LLC, Pensacola, FL, USA), AX3 sensors (Axivity Ltd., Newcastle upon Tyne, UK), Fitbit devices (Fitbit Inc., San Francisco, CA, USA), and GENEActiv devices (Activinsights Ltd., Cambridgeshire, UK). 

Primarily designed for consumer use, these devices integrate various sensors, including inertial measurement units (IMUs)—accelerometers, gyroscopes, and sometimes magnetometers—and bioelectrical sensors. Therefore, their application in clinical settings requires rigorous validation to ensure their accuracy, reliability, and effectiveness. In relation to the importance of acquiring measurements of the use of the UpL within the daily living context of the child, accelerometers are the most used wearable devices to quantify daily-life motor activity in clinical trials and clinical practice [93,94]. A study evaluated accelerometry as a measure of arm movement in children and adolescents with USCP [95].

Furthermore, applying a sensor on either side enables the estimation of laterality, which is particularly relevant for people with unilateral impairments [96]. Indeed, evidence shows that accelerometers can distinguish children with asymmetrical motor impairments [97,98], offering insights into the severity of disability during bimanual real-world activity and potentially guiding therapy selection.

### 4.2. Markerless Systems

Optoelectronic motion capture systems, such as Vicon systems (Oxford Metrics Group, UK), are considered the gold standard for motion analysis and measuring the active range of motion [99]. These systems are highly accurate but are typically limited to structured environments, like clinical laboratories. Wearable sensors, on the other hand, provide a good alternative for monitoring and assessing UpL kinematics in an open or home environment, during the performance of daily-life activities [100]. Moreover, both approaches often require attaching markers or sensors to the body, which can be intrusive and may cause discomfort, particularly for young patients and babies, potentially affecting their natural movements [101].

For these reasons, non-invasive alternatives are being increasingly investigated. Markerless, camera-based systems have emerged as a promising solution due to their unobtrusive nature, ease of deployment, and capacity to capture spontaneous, natural movements in home-based or ecological settings [102]. Crucially, the effectiveness of such systems heavily relies on the performance of advanced software algorithms, which are responsible for the accurate detection and tracking of human movements without the need for physical markers or specialized equipment. Recent applications have shown the potential of markerless video analysis for the pediatric population [103,104,105,106].

Software open source like OpenPose (Carnegie Mellon University, Pittsburgh, PA, USA), MediaPipe (Google LLC, Mountain View, CA, USA), and DeepLabCut [103] (Göttingen University, Göttingen, Germania) allow for the real-time detection of upper limbs, allowing us to obtain the joint kinematics and execution timing. Similarly, alternative non-commercial solutions have been explored based on RGB-D cameras [104,105,106], for quantifying multi-joint coordination and compensatory movement strategies.

### 4.3. Sensorized Objects

Another promising technology for gaining ecological insights into UpL function is the use of sensorized objects. Smart objects, such as sensorized toys, provide a valuable means of monitoring and evaluating children in daily life by capturing detailed information on how they grasp, manipulate, interact with, and release the object. Monitoring these interactions provides a range of metrics that go beyond traditional clinical assessments.

Most studies have focused on the quantitative assessment of early motor skills, particularly grasping and manipulation, demonstrating the feasibility of measuring grasping patterns [107] and forces [108], and the investigation of clinical aspects, such as spatial cognition [109]. The implementation of sensorized objects facilitates the creation of ecologically valid play environments, where instrumented “baby gyms” can capture infants’ spontaneous interactions [110]. By integrating multiple sensors (IMUs and pressure sensors), it is possible to support the early detection of neurological disorders and the longitudinal assessment of grasping development [111]. To overcome the constraints of wired devices, wireless toys were proposed to monitor grasping forces [112]. Cube-based smart toys were designed to detect psychomotor delays [113], and pilot studies confirmed their potential by identifying parameters such as tremor and speed of movements [114]. Multi-toy kits including dolls, cubes, cars, spoons, and balls have also been developed for the objective analysis of play behavior and developmental trajectories [115].

Finally, sensorized objects have been employed as interactive rehabilitation tools. Systems linking toys to computer games encourage children to perform specific movements while collecting clinical data on grip types, movement ranges, and applied forces [116]. More recent platforms also integrate telemonitoring functionalities, supporting autonomous hand rehabilitation and the remote evaluation of progress [117].

### 4.4. Eye-Tracking Systems

Monitoring eye movements and gaze direction can be performed to assess both visual and cognitive function, aspects essential for the effective use of the UpLs. Camera-based eye-tracking systems are a promising technology for this purpose. These systems rely on advanced image processing algorithms and infrared illumination to detect and analyze eye position and pupil dynamics, offering a non-invasive monitoring and assessment system in both experimental and applied settings. Growing evidence indicates that eye-tracking data correlates strongly with traditional cognitive assessment scales, suggesting its effectiveness in assessing visual function (i.e., basic visual processing involved in tasks such as reading [118] and visual–perceptual functions, which involve more complex processes such as visual search [119]), to monitor emotional and cognitive processes (i.e., visual attention, emotional arousal, and stress and cognitive workload) [120] and to assess cognitive and language impairment [121,122] and distractibility in children with neurological disorders [123]. Moreover, due to its portability, this system shows significant potential for use beyond clinical settings, for example, at school [124].

Eye trackers can be classified into two categories: commercial eye trackers and webcam-based eye trackers. The key difference between the two is the type of light used for eye tracking. Commercial eye trackers use infrared light to illuminate the participant’s eyes and analyze the reflections, allowing for greater accuracy. In contrast, webcam-based eye trackers rely on visible light to capture eye movements, typically offering lower precision but at a more affordable cost.

Alongside commercial systems (such as Tobii Technology (Stockholm, Sweden), Smart Eye AB (Gothenburg, Sweden), and Gaze Point (Vancouver, Canada)), webcam-based eye trackers have gained traction, particularly in academic and experimental contexts. Although typically characterized by lower spatial and temporal resolution compared to specialized hardware, these low-cost alternatives offer greater accessibility and scalability, making them suitable for large-scale studies, remote experiments, and preliminary investigations into visual attention and cognitive engagement.

Table 2 summarizes examples of how these technologies have been applied to typically developing (TD) children and those diagnosed with neurological and developmental disorders, to explore their potential applications in evaluating UpL function in children with USCP.

**Table 2:** Promising non-invasive technologies for monitoring and assessing children in daily life.

	ICF	Study Aim	Metrics	Age	P	References
**Wrist-Worn Sensors**	BF	Arm movement	Mean sensitivity, specificity, Youden index	3.4–13.9 y	CP	[99]
	BF	Arm movement	Duration and intensity of movement	2–6 y	TD/USCP	[100]
	BF	Arm movement	Mono-arm use index (MAUI) and bilateral-arm use index (BAUI)	0–17 y	TD/AMD	[101]
	BF	Arm movement	NS	5–12 y	TD/USCP	[102]
**Markerless Systems**	BF/AL	Upper extremity movement assessment	Joint angles, angular trajectories	12–22.6 y	TD/DCP	[103]
	BF/AL	Full-body motion tracking	Max joint angle, smoothness	5–29 y	TD	[104,105]
	BF/AL	Functional assessment of patients with HCP	Joint angle, peak velocity, and peak acceleration	12–17 y	TD	[106]
**Sensorized Objects**	BF/AL	Grasping patterns and manipulation forces	Trial duration, mean, mean and peak pressure, pressure magnitude, pressure frequency, baseline pressure	3 d (SD: 1.5)	TD	[107]
	BF	Monitoring infants’ imitation abilities	Grasping pressure	4–9 m	NS	[108]
	BF/AL	Assessing spatial cognition	Orientation of the block, vertical and horizontal errors, pre-adjustment errors, insertion time, acceleration amplitude	12–36 m	TD	[109]
	BF/AL	Collecting data in a natural play environment to improve the understanding of motor skill development	Maximum toy displacement, time to toy contact, toy contact duration, frequency of toy grasps, mean grasp pressure, mean grasp area	3–11 m	TD	[110]
	BF	Detection of neurological disorders	Grasping pressure	4–9 m	NS	[111]
	BF	Monitoring and measuring infants’ motor development	Maximum and mean pressure, grasping action duration	>37 w	NS	[112]
	BF/AL	Detecting developmental delays	Number, time, and speed of movements; mean of shaking	23–37 m	NS	[113,114]
	BF/AL	Anticipating the diagnosis of autism spectrum disorders, neurodevelopmental disorders, and social fragilities, monitoring children’s ludic behavior	Position and orientation of the toys, time duration of a gesture, features based on accelerometer data, quality of toy manipulation (QoTM)	9–15 m	TD	[115]
	BF/AL	Grip and pinch capability	Range of motion, grip force	3–13 y	TD/MI	[116]
	BF	Telemonitoring rehabilitation progress	Grip force	2–7 y	TD	[117]
**Eye Tracking**	BF	Visual search	Fixation, percentage of gaze points, visual search area	6–12 y	TD/ADHD/CVI/ Dyslexia	[119]
	BF	Language and cognitive assessment	NS	3–15 y	ND	[121]
	BF	Attentional processes	Time in area of interest, press latencies	6–17 y	TD/ADHD/ND	[123]

P = population; NS = not specified; d = days; w = weeks; m = months; y = years; BF = body function and structure level; AL = activity level; CP = cerebral palsy; TD = typical development; DCP = dyskinetic cerebral palsy; HCP = hemiplegic cerebral palsy; ND = neurodevelopment disorder; USCP = unilateral spastic cerebral palsy; AMD = asymmetric motor deficits; ADHD = attention deficit hyperactivity disorder; MI = motor impairments; CVI = cerebral visual impairment.

## 5. Discussion

This narrative review aimed to present an overview of the most frequently used clinical tools that deliver a holistic presentation of the UpL function in children with USCP, while additionally presenting innovative, non-invasive technologies that are promising for assessing children with USCP, for complementary evaluation in daily life (Figure 1). A broad range of assessment tools was presented, tapping diverse functions across the ICF-CY levels, given that the UpL function in children with multifaceted disorders can also be related to non-motor functions [38]. A holistic evaluation allows a more comprehensive understanding of each child’s abilities and limitations, further aiding the formation of tailored care.

For the first research aim, we focused on clinical assessments—including classification systems, performance-based tests, and questionnaires—commonly used to evaluate motor and non-motor functions related to the UpL in children with USCP. The findings indicated that clinical assessments cover a wide range of functioning across the ICF-CY levels. Assessments targeting the ICF-CY Body function level, such as the MAS and two-point discrimination, have been developed for generic populations but are validated for children with USCP. In contrast, available classification systems, clinical tests, and questionnaires that assess UpL function at the ICF-CY Activity level are mainly condition-specific. To date, the clinical evaluation of motor and non-motor abilities comprises the basis of care in CP, with various types of clinical tools serving specific purposes. Classification systems enable uniformity across settings and facilitate multidisciplinary communication, essential in CP [65]. Clinical questionnaires serve as a (time-)efficient way to gain insights into how children function within various contexts. Some can also be filled out by various raters (e.g., self-, parent-, teacher-reported), providing a more differentiated view of the abilities being assessed [125]. Another key strength of clinical questionnaires lies in their ability to capture patients’ lived experience, offering valuable insights that might not be accessible through clinical observation alone. Performance-based clinical assessments are relatively sensitive to changes in performance, making them useful for detecting improvements or declines over time. They are often designed for use in controlled environments, such as clinics or research settings. Standardized instructions further ensure that results remain as comparable as possible across different populations, settings, and examiners [126]. Furthermore, due to their extensive and age-long use, clinical assessments usually have established psychometric properties. Particularly in CP, multiple studies have developed or validated population-specific tools, including normative data, thus increasing the applicability and extraction of robust results [90]. Nonetheless, the literature shows that the psychometric properties of assessments in children with USCP have been examined primarily in relation to UpL function assessments, with less attention given to measures of cognitive function. Yet, the exploration of psychometric properties in neuropsychological assessments is essential. It is also equally important to identify potential accommodations or adaptations of cognitive tests, as these can help account for motor limitations and other condition-specific factors, improving the accuracy and clinical utility of these assessments. Another advantage lies in the clinician–patient interaction during test administration, which enables the real-time observation of the patient’s emotional state or monitoring of compensatory movement strategies, offering deeper insights into performance. This interaction can also enhance motivation by providing feedback in real time. Bartlett and Palisano emphasized that motivation can play a key role in driving motor ability changes in children with CP, regardless of their health condition [127]. By fostering motivation, the results of the evaluation can yield a more accurate representation of a child’s true potential.

Nevertheless, the strict presence of a clinician moderating the evaluation might also work adversely, by creating stress or the feeling of expectation. Furthermore, conducting clinical assessments within a clinical setting may not provide a representative example of the child’s behavior in daily life, as they mainly capture the best of one’s ability or identify the impairment level. Although numerous clinical assessments have a context-specific design, their administration in a controlled environment obscures the unpredictability of daily life. An effective method for collecting accurate information is monitoring the patient within their home and/or natural environment [20], which can be achieved through the implementation of technology.

The second research aim focused on summarizing promising technologies in neurorehabilitation with potential applicability for children with USCP. The findings indicate a wide range of technologies that provide either conventional metrics (e.g., ROM, grip strength) or measures that can only be captured through technology (e.g., angular trajectories, visual search patterns). Such solutions offer objectivity, reducing reliance on bias-mediated observation. They further enhance ecological validity by capturing real-world patient behavior in daily life, providing insights that traditional clinical tests may not capture. They also ensure consistency and reproducibility, allowing standardized assessments to be reliably repeated across time and settings. In addition, their minimal invasiveness improves patient comfort, particularly in pediatric populations such as children with USCP, while their high portability enables use beyond laboratories, making these tools accessible in clinics, in schools, or even at home. Collectively, these advantages enrich the quality of clinical data, enable the more precise monitoring of functional abilities, and create opportunities for integrating rehabilitation into daily contexts.

An interesting finding was that the use of technology can mainly provide information across the ICF-CY Body Function and Structure and Activity levels. These levels are more easily addressed through technological solutions, since the ICF-CY levels of Participation and Quality of Life require a contextual understanding of the patient’s lived experience, occasionally relying on self-reported measures that provide a subjective point of view. Yet, strategies combining methods could be explored to extend the application of technology in these domains. Additionally, many systems face challenges with user acceptability, particularly wrist-worn sensors that require consistent use or charging [128], compromising compliance. With respect to camera-based technologies, environmental factors, such as lighting or background conditions, can affect the accuracy of markerless and eye-tracking systems. Particularly for eye tracking, physical characteristics such as eye color or the use of prescription glasses can further decrease the quality of the collected data. Sensorized objects and toys are often limited by their reliance on a single device or small number of devices [129]. The development of larger sets of objects increases the costs and customization required. Lastly, most studies on promising technologies remain proof-of-concept in TD children, and only a few studies include individuals with motor impairments, making clinical validation difficult. There is, therefore, the need for thorough validation and co-design with clinicians to ensure clinical applicability in other populations.

A careful integration of established clinical assessments with promising technologies, while taking into account their respective limitations, can improve rehabilitation outcomes by combining their strengths. For instance, wrist-worn sensors offer a minimally invasive means of accurately assessing asymmetries in UpL use at the Activity level of the ICF-CY. These sensors can also be integrated into performance-based clinical tools, such as the AHA or the BBT, providing complementary information on UpL spatial trajectories during test execution. Due to their high portability, markerless [130] systems can be used for the tracking of mirror movements, asymmetries between the affected and less-affected hand, or movement smoothness, making them able to assess modalities across the ICF-CY levels of Body Function and Structure and Activity. Additionally, the ability to use these systems both in real time and offline makes them an excellent means for assisting video-based assessments of the UpL. Combined with trained machine learning algorithms, markerless systems could potentially be used in the scoring of video-based assessments, such as the AHA or the MA2, [130], reducing the need for the clinician to watch the whole video session for assessment. Sensorized objects offer also the potential to complement the current clinical assessments. For example, in the context of ICF-CY Body Function, grip strength is assessed through the Jamar Dynamometer, which is widely considered the gold standard for measuring grip strength in children with CP [30]. However, its use requires a standardized testing position, minimizing ecological insights into grip strength during diverse arm positions. Thus, sensorized objects, such as sensorized toys, could overcome this gap, by monitoring and quantifying the child’s interaction with it. Additionally, sensorized toys equipped with IMUs can provide valuable data on movement patterns, allowing clinicians to monitor motor abilities more precisely, extending their use also for the ICF-CY Activity level. Shape-matching tasks that are object-based can also be equipped with sensors that can quantify task performance parameters, such as time for task completion or object trajectory, allowing the evaluation of UpL activity and cognitive-related functions. Lastly, eye tracking can primarily assess functions at the ICF-CY Body Function and Structure level. One example is its integration into attention tests. Traditional sustained attention assessments, such as continuous performance tests, rely on motor responses, which can be limited by motor impairments and provide limited real-world performance. Eye tracking offers a complementary approach by capturing objective, real-time measures of gaze behavior—including microsaccades and pupil dilations—allowing the detection of lapses in attention [131] even when button-press responses are correct, using only a single camera.

We acknowledge that, due to the narrative nature of this methodology, some clinically relevant tools may not have been captured. While selection was guided by the authors’ consensus and informed by the literature, this approach does not aim to be exhaustive. Instead, it reflects a focused synthesis of widely used assessments for children over the age of 5, and there remains the possibility that other valuable tools—particularly those emerging in specific clinical contexts or less frequently reported in the literature—were not included. Future work should aim to build on this narrative synthesis by conducting more systematic or scoping reviews that comprehensively map the full range of available clinical assessment tools for children with USCP.

## 6. Future Directions

As research progresses, technological solutions are likely to play an increasingly central role in clinical practice, supporting more personalized and accessible rehabilitation strategies for children with CP. To realize this potential, pilot programs and interdisciplinary collaborations between clinicians, researchers, and technologists will be essential. Such collaborations can facilitate the development and validation of innovative technological solutions that complement and extend current clinical assessments, while carefully addressing their known limitations. The establishment of integration frameworks will help to ensure that technologies translate into scientifically sound, clinically feasible, and meaningful solutions for the clinical reality. Additionally, studies implementing these technologies in specific populations are essential to explore their psychometric properties and clinical utility.

## 7. Conclusions

This study highlights the value of a comprehensive approach to evaluating UpL function in children with USCP, given the multidimensional nature of the condition. Traditional assessments, classification systems, and questionnaires remain the standard evaluation approach due to their established psychometric properties, ability to capture patient perspectives, and facilitation of multidisciplinary communication. Integrating these clinical assessments with emerging, non-invasive technologies—such as wearable sensors, markerless motion capture, sensorized objects, and eye-tracking systems—allows for the assessment of both motor and non-motor functions in real-world contexts, providing ecological insights that can inform tailored rehabilitation strategies. While promising, these technologies cannot replace established clinical tools, but their use as an adjunct is encouraged, offering metrics inaccessible to traditional tests as well as ecologically valid data. Key gaps remain the lack of USCP-specific psychometric validation for most neuropsychological tests, and the need to accommodate neuropsychological assessments to condition-specific requirements. For promising technologies, notable gaps remain the limited coverage of ICF-CY Participation and Quality of Life domains, which restricts their ability to capture patient-centered outcomes. In addition, there is a scarcity of studies involving children with motor impairments, limiting evidence on the applicability, feasibility, and validity of these technologies in clinical populations.

## Data Availability

No new data were created or analyzed in this study. Data sharing is not applicable to this article.

## References

[1] McIntyre S., Goldsmith S., Webb A., Ehlinger V., Hollung S.J., McConnell K., Arnaud C., Smithers-Sheedy H., Oskoui M., Khandaker G. (2022). Global Prevalence of Cerebral Palsy: A Systematic Analysis. Dev. Med. Child. Neurol..

[2] Wimalasundera N., Stevenson V.L. (2016). Cerebral Palsy. Pract. Neurol..

[3] Beckung E., Hagberg G., Uldall P., Cans C. (2008). Probability of Walking in Children with Cerebral Palsy in Europe. Pediatrics.

[4] Klingels K., Demeyere I., Jaspers E., De Cock P., Molenaers G., Boyd R., Feys H. (2012). Upper Limb Impairments and Their Impact on Activity Measures in Children with Unilateral Cerebral Palsy. Eur. J. Paediatr. Neurol..

[5] Hung Y.-C., Charles J., Gordon A.M. (2004). Bimanual Coordination during a Goal-Directed Task in Children with Hemiplegic Cerebral Palsy. Dev. Med. Child. Neurol..

[6] Mailleux L., Klingels K., Fiori S., Simon-Martinez C., Demaerel P., Locus M., Fosseprez E., Boyd R.N., Guzzetta A., Ortibus E. (2017). How Does the Interaction of Presumed Timing, Location and Extent of the Underlying Brain Lesion Relate to Upper Limb Function in Children with Unilateral Cerebral Palsy?. Eur. J. Paediatr. Neurol..

[7] Simon-Martinez C., Jaspers E., Mailleux L., Ortibus E., Klingels K., Wenderoth N., Feys H. (2018). Corticospinal Tract Wiring and Brain Lesion Characteristics in Unilateral Cerebral Palsy: Determinants of Upper Limb Motor and Sensory Function. Neural Plast..

[8] Guzzetta A., Fazzi B., Mercuri E., Bertuccelli B., Canapicchi R., van Hof-van Duin J., Cioni G. (2001). Visual Function in Children with Hemiplegia in the First Years of Life. Dev. Med. Child. Neurol..

[9] Abgottspon S., Steiner L., Slavova N., Steinlin M., Grunt S., Everts R. (2022). Relationship between Motor Abilities and Executive Functions in Patients after Pediatric Stroke. Appl. Neuropsychol. Child..

[10] Rose S., Guzzetta A., Pannek K., Boyd R. (2011). MRI Structural Connectivity, Disruption of Primary Sensorimotor Pathways, and Hand Function in Cerebral Palsy. Brain Connect..

[11] Hoon A.H., Stashinko E., Nagae L.M., Lin D.D., Keller J., Bastian A., Campbell M.L., Levey E., Mori S., Johnston M.V. (2009). Sensory and Motor Deficits in Children with Cerebral Palsy Born Preterm Correlate with Diffusion Tensor Imaging Abnormalities in Thalamocortical Pathways. Dev. Med. Child. Neurol..

[12] Basu A.P., Pearse J., Kelly S., Wisher V., Kisler J. (2015). Early Intervention to Improve Hand Function in Hemiplegic Cerebral Palsy. Front. Neurol..

[13] Auld M.L., Boyd R.N., Moseley G.L., Ware R.S., Johnston L.M. (2012). Impact of Tactile Dysfunction on Upper-Limb Motor Performance in Children with Unilateral Cerebral Palsy. Arch. Phys. Med. Rehabil..

[14] Bleyenheuft Y., Gordon A.M. (2013). Precision Grip Control, Sensory Impairments and Their Interactions in Children with Hemiplegic Cerebral Palsy: A Systematic Review. Res. Dev. Disabil..

[15] James S., Ziviani J., Ware R.S., Boyd R.N. (2015). Relationships between Activities of Daily Living, Upper Limb Function, and Visual Perception in Children and Adolescents with Unilateral Cerebral Palsy. Dev. Med. Child. Neurol..

[16] Kalkantzi A., Kleeren L., Baeyens D., Decraene L., Crotti M., Klingels K., Van Campenhout A., Verheyden G., Ortibus E., Feys H. (2025). Daily-life Executive Functions and Bimanual Performance in Children with Unilateral Cerebral Palsy. Dev. Med. Child. Neurol..

[17] World Health Organization (2007). International Classification of Functioning, Disability and Health: Children & Youth Version: ICF-CY.

[18] Bland M.D., Sturmoski A., Whitson M., Harris H., Connor L.T., Fucetola R., Edmiaston J., Huskey T., Carter A., Kramper M. (2013). Clinician Adherence to a Standardized Assessment Battery Across Settings and Disciplines in a Poststroke Rehabilitation Population. Arch. Phys. Med. Rehabil..

[19] Carcreff L., Gerber C.N., Paraschiv-Ionescu A., De Coulon G., Newman C.J., Aminian K., Armand S. (2020). Comparison of gait characteristics between clinical and daily life settings in children with cerebral palsy. Sci. Rep..

[20] van der Lee J.H., Beckerman H., Knol D.L., de Vet H.C.W., Bouter L.M. (2004). Clinimetric Properties of the Motor Activity Log for the Assessment of Arm Use in Hemiparetic Patients. Stroke.

[21] Rozaire J., Paquin C., Henry L., Agopyan H., Bard-Pondarré R., Naaim A., Duprey S., Chaleat-Valayer E. (2024). A Systematic Review of Instrumented Assessments for Upper Limb Function in Cerebral Palsy: Current Limitations and Future Directions. J. Neuroeng. Rehabil..

[22] Cacioppo M., Loos A., Lempereur M., Brochard S. (2023). Bimanual Movements in Children with Cerebral Palsy: A Systematic Review of Instrumented Assessments. J. Neuroeng. Rehabil..

[23] Boroumand S., Pashmdarfard M., Hamedi D., Mehraban A.H. (2025). Clinical Tools for Assessing One-Handed Skills in Children with Cerebral Palsy: An Umbrella Review. Occup. Ther. Int..

[24] Mia M.R., Ahamed S.I., Fial A., Nemanich S. (2024). A Scoping Review on Mobile Health Technology for Assessment and Intervention of Upper Limb Motor Function in Children with Motor Impairments. Games Health J..

[25] Mokkink L.B., Prinsen C.A.C., Bouter L.M., Vet H.C.W.d., Terwee C.B. (2016). The COnsensus-Based Standards for the Selection of Health Measurement INstruments (COSMIN) and How to Select an Outcome Measurement Instrument. Braz. J. Phys. Ther..

[26] Klingels K., De Cock P., Molenaers G., Desloovere K., Huenaerts C., Jaspers E., Feys H. (2010). Upper Limb Motor and Sensory Impairments in Children with Hemiplegic Cerebral Palsy. Can They Be Measured Reliably?. Disabil. Rehabil..

[27] Fedrizzi E., Pagliano E., Andreucci E., Oleari G. (2003). Hand Function in Children with Hemiplegic Cerebral Palsy: Prospective Follow-up and Functional Outcome in Adolescence. Dev. Med. Child. Neurol..

[28] Harb A., Margetis K., Kishner S. (2024). Modified Ashworth Scale.

[29] Nourizadeh M., Shadgan B., Abbasidezfouli S., Juricic M., Mulpuri K. (2024). Methods of Muscle Spasticity Assessment in Children with Cerebral Palsy: A Scoping Review. J. Orthop. Surg. Res..

[30] Dekkers K.J.F.M., Rameckers E.A.A., Smeets R.J.E.M., Janssen-Potten Y.J.M. (2014). Upper Extremity Strength Measurement for Children with Cerebral Palsy: A Systematic Review of Available Instruments. Phys. Ther..

[31] Magne V.A., Adde L., Hoare B., Klingels K., Simon-Martinez C., Mailleux L., Lydersen S., Elvrum A.G. (2021). Assessment of Mirror Movements in Children and Adolescents with Unilateral Cerebral Palsy: Reliability of the Woods and Teuber Scale. Dev. Med. Child. Neurol..

[32] Bumin G., Kavak S.T. (2008). An Investigation of the Factors Affecting Handwriting Performance in Children with Hemiplegic Cerebral Palsy. Disabil. Rehabil..

[33] Klingels K., Jaspers E., Van de Winckel A., De Cock P., Molenaers G., Feys H. (2010). A Systematic Review of Arm Activity Measures for Children with Hemiplegic Cerebral Palsy. Clin. Rehabil..

[34] Auld M.L., Boyd R.N., Moseley G.L., Johnston L.M. (2011). Tactile Assessment in Children with Cerebral Palsy: A Clinimetric Review. Phys. Occup. Ther. Pediatr..

[35] Fazzi E., Signorini S.G., LA Piana R., Bertone C., Misefari W., Galli J., Balottin U., Bianchi P.E. (2012). Neuro-ophthalmological Disorders in Cerebral Palsy: Ophthalmological, Oculomotor, and Visual Aspects. Dev. Med. Child. Neurol..

[36] Jacobson L., Rydberg A., Eliasson A., Kits A., Flodmark O. (2010). Visual Field Function in School-aged Children with Spastic Unilateral Cerebral Palsy Related to Different Patterns of Brain Damage. Dev. Med. Child. Neurol..

[37] Crotti M., Ortibus E., Mailleux L., Decraene L., Kleeren L., Itzhak N. (2024). Ben Visual, Perceptual Functions, and Functional Vision in Children with Unilateral Cerebral Palsy Compared to Children with Neurotypical Development. Dev. Med. Child. Neurol..

[38] Crotti M., Ortibus E., Ben Itzhak N., Kleeren L., Decraene L., Leenaerts N., Feys H., Mailleux L. (2024). The Relation between Visual Functions, Functional Vision, and Bimanual Function in Children with Unilateral Cerebral Palsy. Res. Dev. Disabil..

[39] Auld M., Boyd R., Moseley G.L., Johnston L. (2011). Seeing the Gaps: A Systematic Review of Visual Perception Tools for Children with Hemiplegia. Disabil. Rehabil..

[40] Stadskleiv K. (2020). Cognitive Functioning in Children with Cerebral Palsy. Dev. Med. Child. Neurol..

[41] Stadskleiv K., Jahnsen R., Andersen G.L., von Tetzchner S. (2017). Executive Functioning in Children Aged 6–18 Years with Cerebral Palsy. J. Dev. Phys. Disabil..

[42] Bodimeade H.L., Whittingham K., Lloyd O., Boyd R.N. (2013). Executive Function in Children and Adolescents with Unilateral Cerebral Palsy. Dev. Med. Child. Neurol..

[43] Bottcher L., Flachs E.M., Uldall P. (2010). Attentional and Executive Impairments in Children with Spastic Cerebral Palsy. Dev. Med. Child. Neurol..

[44] Friedman N.P., Miyake A. (2017). Unity and Diversity of Executive Functions: Individual Differences as a Window on Cognitive Structure. Cortex.

[45] Wechsler D. (2012). Wechsler Preschool and Primary Scale of Intelligence.

[46] Wechsler D. (2014). Wechsler Intelligence Scale for Children.

[47] Canivez G.L., McGill R.J., Dombrowski S.C., Watkins M.W., Pritchard A.E., Jacobson L.A. (2020). Construct Validity of the WISC-V in Clinical Cases: Exploratory and Confirmatory Factor Analyses of the 10 Primary Subtests. Assessment.

[48] Gioia G.A., Isquith P.K., Guy S.C., Kenworthy L. (2000). TEST REVIEW Behavior Rating Inventory of Executive Function. Child Neuropsychol..

[49] Pereira A., Lopes S., Magalhães P., Sampaio A., Chaleta E., Rosário P. (2018). How Executive Functions Are Evaluated in Children and Adolescents with Cerebral Palsy? A Systematic Review. Front. Psychol..

[50] Shaked D., Faulkner L.M.D., Tolle K., Wendell C.R., Waldstein S.R., Spencer R.J. (2020). Reliability and Validity of the Conners’ Continuous Performance Test. Appl. Neuropsychol. Adult.

[51] Miyake A., Friedman N.P., Emerson M.J., Witzki A.H., Howerter A., Wager T.D. (2000). The Unity and Diversity of Executive Functions and Their Contributions to Complex “Frontal Lobe” Tasks: A Latent Variable Analysis. Cogn. Psychol..

[52] Chagas P.S.C., Magalhães E.D.D., Sousa Junior R.R., Romeros A.C.S.F., Palisano R.J., Leite H.R., Rosenbaum P. (2023). Development of Children, Adolescents, and Young Adults with Cerebral Palsy According to the ICF: A Scoping Review. Dev. Med. Child. Neurol..

[53] Eliasson A.C., Krumlinde-Sundholm L., Rösblad B., Beckung E., Arner M., Öhrvall A.M., Rosenbaum P. (2006). The Manual Ability Classification System (MACS) for Children with Cerebral Palsy: Scale Development and Evidence of Validity and Reliability. Dev. Med. Child. Neurol..

[54] Elvrum A.-K.G., Andersen G.L., Himmelmann K., Beckung E., Öhrvall A.-M., Lydersen S., Vik T. (2016). Bimanual Fine Motor Function (BFMF) Classification in Children with Cerebral Palsy: Aspects of Construct and Content Validity. Phys. Occup. Ther. Pediatr..

[55] House J.H., Gwathmey F.W., Fidler M.O. (1981). A Dynamic Approach to the Thumb-in Palm Deformity in Cerebral Palsy. J. Bone Jt. Surg. Am..

[56] Geerdink Y., Lindeboom R., de Wolf S., Steenbergen B., Geurts A.C.H., Aarts P. (2014). Assessment of Upper Limb Capacity in Children with Unilateral Cerebral Palsy: Construct Validity of a Rasch-reduced Modified House Classification. Dev. Med. Child. Neurol..

[57] Krumlinde-Sundholm L. (2003). Development of the Assisting Hand Assessment: A Rasch-built measure intended for children with unilateral upper limb impairments. Scand. J. Occup. Ther..

[58] Randall M., Imms C., Carey L.M., Pallant J.F. (2014). Rasch Analysis of The Melbourne Assessment of Unilateral Upper Limb Function. Dev. Med. Child. Neurol..

[59] Gerber C.N., Plebani A., Labruyère R. (2019). Translation, Reliability, and Clinical Utility of the Melbourne Assessment 2. Disabil. Rehabil..

[60] Araneda R., Ebner-Karestinos D., Paradis J., Saussez G., Friel K.M., Gordon A.M., Bleyenheuft Y. (2019). Reliability and Responsiveness of the Jebsen-Taylor Test of Hand Function and the Box and Block Test for Children with Cerebral Palsy. Dev. Med. Child. Neurol..

[61] Platz T., Pinkowski C., van Wijck F., Kim I.-H., di Bella P., Johnson G. (2005). Reliability and Validity of Arm Function Assessment with Standardized Guidelines for the Fugl-Meyer Test, Action Research Arm Test and Box and Block Test: A Multicentre Study. Clin. Rehabil..

[62] Arnould C., Penta M., Renders A., Thonnard J.-L. (2004). ABILHAND-Kids. Neurology.

[63] Pearse J., Basu A.P. (2017). ABILHAND-Kids Questionnaire: Responsive to Change or Room for Change?. Dev. Med. Child. Neurol..

[64] Charles J.R., Wolf S.L., Schneider J.A., Gordon A.M. (2006). Efficacy of a Child-Friendly Form of Constraint-Induced Movement Therapy in Hemiplegic Cerebral Palsy: A Randomized Control Trial. Dev. Med. Child. Neurol..

[65] Paulson A., Vargus-Adams J. (2017). Overview of Four Functional Classification Systems Commonly Used in Cerebral Palsy. Children.

[66] James S., Ziviani J., Boyd R. (2014). A Systematic Review of Activities of Daily Living Measures for Children and Adolescents with Cerebral Palsy. Dev. Med. Child. Neurol..

[67] James S., Ziviani J., Ware R.S., Boyd R.N. (2016). Test–Retest Reproducibility of the Assessment of Motor and Process Skills in Children with Unilateral Cerebral Palsy. Phys. Occup. Ther. Pediatr..

[68] Coster W., Law M., Bedell G., Khetani M., Cousins M., Teplicky R. (2012). Development of the Participation and Environment Measure for Children and Youth: Conceptual Basis. Disabil. Rehabil..

[69] Eiser C., Morse R. (2001). Quality-of-Life Measures in Chronic Diseases of Childhood. Health Technol. Assess..

[70] Wilson I.B. (1995). Cleary, P.D. Linking Clinical Variables with Health-Related Quality of Life. A Conceptual Model of Patient Outcomes. JAMA: J. Am. Med. Assoc..

[71] Makris T., Dorstyn D., Crettenden A. (2021). Quality of Life in Children and Adolescents with Cerebral Palsy: A Systematic Review with Meta-Analysis. Disabil. Rehabil..

[72] Mpundu-Kaambwa C., Chen G., Huynh E., Russo R., Ratcliffe J. (2018). A Review of Preference-Based Measures for the Assessment of Quality of Life in Children and Adolescents with Cerebral Palsy. Qual. Life Res..

[73] Auld M.L., Ware R.S., Boyd R.N., Moseley G.L., Johnston L.M. (2012). Reproducibility of Tactile Assessments for Children with Unilateral Cerebral Palsy. Phys. Occup. Ther. Pediatr..

[74] Dua K., Lancaster T.P., Abzug J.M. (2019). Age-Dependent Reliability of Semmes-Weinstein and 2-Point Discrimination Tests in Children. J. Pediatr. Orthop..

[75] Syeda M.M., Climie E.A. (2014). Test Review: Wechsler Preschool and Primary Scale of Intelligence–Fourth Edition. J. Psychoeduc. Assess..

[76] Andrikopoulos V. (2021). Exploring the Validity and Reliability of the WISC-IV: A Review of the Literature. J. Soc. Sci. Stud..

[77] Piovesana A.M., Ross S., Whittingham K., Ware R.S., Boyd R.N. (2015). Stability of Executive Functioning Measures in 8–17-Year-Old Children with Unilateral Cerebral Palsy. Clin. Neuropsychol..

[78] Orsini A. (1994). Corsi’s Block-Tapping Test: Standardization and Concurrent Validity with WISC—R for Children Aged 11 to 16. Percept. Mot. Ski..

[79] Krumlinde-Sundholm L., Holmefur M., Kottorp A., Eliasson A.C. (2007). The Assisting Hand Assessment: Current evidence of validity, reliability, and responsiveness to change. Dev. Med. Child Neurol..

[80] Holmefur M.M., Krumlinde-Sundholm L. (2016). Psychometric Properties of a Revised Version of the Assisting Hand Assessment (Kids-AHA 5.0). Dev. Med. Child. Neurol..

[81] Louwers A., Beelen A., Holmefur M., Krumlinde-Sundholm L. (2016). Development of the Assisting Hand Assessment for Adolescents (Ad-AHA) and Validation of the AHA from 18 Months to 18 Years. Dev. Med. Child. Neurol..

[82] Wang T.-N., Liang K.-J., Liu Y.-C., Shieh J.-Y., Chen H.-L. (2017). Psychometric and Clinimetric Properties of the Melbourne Assessment 2 in Children with Cerebral Palsy. Arch. Phys. Med. Rehabil..

[83] Sköld A., Hermansson L.N., Krumlinde-Sundholm L., Eliasson A.C. (2011). Development and Evidence of Validity for the Children’s Hand-Use Experience Questionnaire (CHEQ). Dev. Med. Child. Neurol..

[84] Amer A., Eliasson A., Peny-Dahlstrand M., Hermansson L. (2016). Validity and Test–Retest Reliability of Children’s Hand-use Experience Questionnaire in Children with Unilateral Cerebral Palsy. Dev. Med. Child. Neurol..

[85] Mitchell L.E., Ziviani J., Boyd R.N. (2015). Characteristics Associated with Physical Activity among Independently Ambulant Children and Adolescents with Unilateral Cerebral Palsy. Dev. Med. Child. Neurol..

[86] Kang M., Smith E., Goldsmith C.H., Switzer L., Rosenbaum P., Wright F.V., Fehlings D. (2020). Documenting Change with the Canadian Occupational Performance Measure for Children with Cerebral Palsy. Dev. Med. Child. Neurol..

[87] Sakzewski L., Boyd R., Ziviani J. (2007). Clinimetric Properties of Participation Measures for 5- to 13-year-old Children with Cerebral Palsy: A Systematic Review. Dev. Med. Child. Neurol..

[88] Coster W., Bedell G., Law M., Khetani M.A., Teplicky R., Liljenquist K., Gleason K., Kao Y.-C. (2011). Psychometric Evaluation of the Participation and Environment Measure for Children and Youth. Dev. Med. Child. Neurol..

[89] Davis E., Shelly A., Waters E., Davern M. (2010). Measuring the Quality of Life of Children with Cerebral Palsy: Comparing the Conceptual Differences and Psychometric Properties of Three Instruments. Dev. Med. Child. Neurol..

[90] Carlon S., Shields N., Yong K., Gilmore R., Sakzewski L., Boyd R. (2010). A Systematic Review of the Psychometric Properties of Quality of Life Measures for School Aged Children with Cerebral Palsy. BMC Pediatr..

[91] Waters E., Davis E., Mackinnon A., Boyd R., Graham H.K., Kai Lo S., Wolfe R., Stevenson R., Bjornson K., Blair E. (2007). Psychometric Properties of the Quality of Life Questionnaire for Children with CP. Dev. Med. Child. Neurol..

[92] Braito I., Maselli M., Sgandurra G., Inguaggiato E., Beani E., Cecchi F., Cioni G., Boyd R. (2018). Assessment of Upper Limb Use in Children with Typical Development and Neurodevelopmental Disorders by Inertial Sensors: A Systematic Review. J. Neuroeng. Rehabil..

[93] Cervantes C.M., Porretta D.L. (2010). Physical Activity Measurement Among Individuals with Disabilities: A Literature Review. Adapt. Phys. Act. Q..

[94] Wang Q., Markopoulos P., Yu B., Chen W., Timmermans A. (2017). Interactive Wearable Systems for Upper Body Rehabilitation: A Systematic Review. J. Neuroeng. Rehabil..

[95] Dawe J., Yang J.F., Fehlings D., Likitlersuang J., Rumney P., Zariffa J., Musselman K.E. (2019). Validating Accelerometry as a Measure of Arm Movement for Children with Hemiplegic Cerebral Palsy. Phys. Ther..

[96] Sokal B., Uswatte G., Vogtle L., Byrom E., Barman J. (2015). Everyday Movement and Use of the Arms: Relationship in Children with Hemiparesis Differs from Adults. J. Pediatr. Rehabil. Med..

[97] Hoyt C.R., Van A.N., Ortega M., Koller J.M., Everett E.A., Nguyen A.L., Lang C.E., Schlaggar B.L., Dosenbach N.U.F. (2019). Detection of Pediatric Upper Extremity Motor Activity and Deficits with Accelerometry. JAMA Netw. Open.

[98] Srinivasan S., Amonkar N., Kumavor P.D., Bubela D. (2024). Measuring Upper Extremity Activity of Children with Unilateral Cerebral Palsy Using Wrist-Worn Accelerometers: A Pilot Study. Am. J. Occup. Ther..

[99] Francisco-Martínez C., Prado-Olivarez J., Padilla-Medina J.A., Díaz-Carmona J., Pérez-Pinal F.J., Barranco-Gutiérrez A.I., Martínez-Nolasco J.J. (2021). Upper Limb Movement Measurement Systems for Cerebral Palsy: A Systematic Literature Review. Sensors.

[100] Walmsley C.P., Williams S.A., Grisbrook T., Elliott C., Imms C., Campbell A. (2018). Measurement of Upper Limb Range of Motion Using Wearable Sensors: A Systematic Review. Sports Med. Open.

[101] Khan M.H., Zöller M., Farid M.S., Grzegorzek M. (2020). Marker-Based Movement Analysis of Human Body Parts in Therapeutic Procedure. Sensors.

[102] Metcalf C.D., Robinson R., Malpass A.J., Bogle T.P., Dell T.A., Harris C., Demain S.H. (2013). Markerless Motion Capture and Measurement of Hand Kinematics: Validation and Application to Home-Based Upper Limb Rehabilitation. IEEE Trans. Biomed. Eng..

[103] Vanmechelen I., Van Wonterghem E., Aerts J.-M., Hallez H., Desloovere K., Van de Walle P., Buizer A.I., Monbaliu E., Haberfehlner H. (2024). Markerless Motion Analysis to Assess Reaching-Sideways in Individuals with Dyskinetic Cerebral Palsy: A Validity Study. J. Biomech..

[104] Hesse N., Baumgartner S., Gut A., van Hedel H.J.A. (2023). Concurrent Validity of a Custom Method for Markerless 3D Full-Body Motion Tracking of Children and Young Adults Based on a Single RGB-D Camera. IEEE Trans. Neural Syst. Rehabil. Eng..

[105] Hesse N., Baumgartner S., Gut A., Van Hedel H.J.A. (2024). Concurrent Validity of Motion Parameters Measured With an RGB-D Camera-Based Markerless 3D Motion Tracking Method in Children and Young Adults. IEEE J. Transl. Eng. Health Med..

[106] Rammer J.R., Krzak J.J., Riedel S.A., Harris G.F. (2014). Evaluation of Upper Extremity Movement Characteristics during Standardized Pediatric Functional Assessment with a Kinect-Based Markerless Motion Analysis System. Proceedings of the 2014 36th Annual International Conference of the IEEE Engineering in Medicine and Biology Society.

[107] Molina M., Jouen F. (1998). Modulation of the Palmar Grasp Behavior in Neonates According to Texture Property. Infant. Behav. Dev..

[108] Baldoli I., Cecchi F., Guzzetta A., Laschi C. (2015). Sensorized Graspable Devices for the Study of Motor Imitation in Infants. Proceedings of the 2015 37th Annual International Conference of the IEEE Engineering in Medicine and Biology Society (EMBC).

[109] Campolo D., Taffoni F., Formica D., Iverson J., Sparaci L., Keller F., Guglielmelli E. (2012). Embedding Inertial-Magnetic Sensors in Everyday Objects: Assessing Spatial Cognition in Children. J. Integr. Neurosci..

[110] Goyal V., Torres W., Rai R., Shofer F., Bogen D., Bryant P., Prosser L., Johnson M.J. (2017). Quantifying Infant Physical Interactions Using Sensorized Toys in a Natural Play Environment. Proceedings of the 2017 International Conference on Rehabilitation Robotics (ICORR).

[111] Cecchi F., Serio S.M., Del Maestro M., Laschi C., Sgandurra G., Cioni G., Dario P. (2010). Design and Development of “Biomechatronic Gym” for Early Detection of Neurological Disorders in Infants. Proceedings of the 2010 Annual International Conference of the IEEE Engineering in Medicine and Biology.

[112] Serio S.M., Cecchi F., Assaf T., Laschi C., Dario P. (2013). Design and Development of a Sensorized Wireless Toy for Measuring Infants’ Manual Actions. IEEE Trans. Neural Syst. Rehabil. Eng..

[113] Rivera D., García A., Alarcos B., Velasco J., Ortega J., Martínez-Yelmo I. (2016). Smart Toys Designed for Detecting Developmental Delays. Sensors.

[114] Gutiérrez García M.A., Martín Ruiz M.L., Rivera D., Vadillo L., Valero Duboy M.A. (2017). A Smart Toy to Enhance the Decision-Making Process at Children’s Psychomotor Delay Screenings: A Pilot Study. J. Med. Internet Res..

[115] Faraci F.D., Papandrea M., Puiatti A., Agustoni S., Giulivi S., DrApuzzo V., Giordano S., Righi F., Barberis O., Thommen E. (2018). AutoPlay: A Smart Toys-Kit for an Objective Analysis of Children Ludic Behavior and Development. Proceedings of the 2018 IEEE International Symposium on Medical Measurements and Applications (MeMeA).

[116] Boschi S.R.M.S., Frère A.F. (2013). Grip and Pinch Capability Assessment System for Children. Med. Eng. Phys..

[117] Borghese N.A., Essenziale J., Mainetti R., Mancon E., Pagliaro R., Pajardi G. (2019). Hand Rehabilitation and Telemonitoring through Smart Toys. Sensors.

[118] Lampe R., Turova V., Blumenstein T., Alves-Pinto A. (2014). Eye Movement during Reading in Young Adults with Cerebral Palsy Measured with Eye Tracking. Postgrad. Med..

[119] Hokken M.J., Stein N., Pereira R.R., Rours I.G.I.J.G., Frens M.A., van der Steen J., Pel J.J.M., Kooiker M.J.G. (2024). Eyes on CVI: Eye Movements Unveil Distinct Visual Search Patterns in Cerebral Visual Impairment Compared to ADHD, Dyslexia, and Neurotypical Children. Res. Dev. Disabil..

[120] Skaramagkas V., Giannakakis G., Ktistakis E., Manousos D., Karatzanis I., Tachos N., Tripoliti E., Marias K., Fotiadis D.I., Tsiknakis M. (2023). Review of Eye Tracking Metrics Involved in Emotional and Cognitive Processes. IEEE Rev. Biomed. Eng..

[121] Tao L., Wang Q., Liu D., Wang J., Zhu Z., Feng L. (2020). Eye Tracking Metrics to Screen and Assess Cognitive Impairment in Patients with Neurological Disorders. Neurol. Sci..

[122] Graziola F., Garone G., Grasso M., Capuano A. (2021). Cognitive Assessment in GNAO1 Neurodevelopmental Disorder Using an Eye Tracking System. J. Clin. Med..

[123] Sweere D.J.J., Pel J.J.M., Kooiker M.J.G., van Dijk J.P., van Gemert E.J.J.M., Hurks P.P.M., Klinkenberg S., Vermeulen R.J., Hendriksen J.G.M. (2022). Clinical Utility of Eye Tracking in Assessing Distractibility in Children with Neurological Disorders or ADHD: A Cross-Sectional Study. Brain Sci..

[124] Bolden D., Barmby P., Raine S., Gardner M. (2015). How Young Children View Mathematical Representations: A Study Using Eye-Tracking Technology. Educ. Res..

[125] Marshall G. (2005). The Purpose, Design and Administration of a Questionnaire for Data Collection. Radiography.

[126] Javvaji C.K., Vagha J.D., Meshram R.J., Taksande A. (2023). Assessment Scales in Cerebral Palsy: A Comprehensive Review of Tools and Applications. Cureus.

[127] Bartlett D.J., Palisano R.J. (2002). Physical Therapists’ Perceptions of Factors Influencing the Acquisition of Motor Abilities of Children with Cerebral Palsy: Implications for Clinical Reasoning. Phys. Ther..

[128] Rast F.M., Herren S., Labruyère R. (2022). Acceptability of Wearable Inertial Sensors, Completeness of Data, and Day-to-Day Variability of Everyday Life Motor Activities in Children and Adolescents with Neuromotor Impairments. Front. Rehabil. Sci..

[129] De Luca G., Bressi A., Maselli M., Greco F., Cianchetti M. (2024). Sensorizing Objects with Soft and Flexible Sensors Based on Laser-Induced Graphene. Proceedings of the 2024 46th Annual International Conference of the IEEE Engineering in Medicine and Biology Society (EMBC).

[130] Haberfehlner H., Roth Z., Vanmechelen I., Buizer A.I., Jeroen Vermeulen R., Koy A., Aerts J.-M., Hallez H., Monbaliu E. (2024). A Novel Video-Based Methodology for Automated Classification of Dystonia and Choreoathetosis in Dyskinetic Cerebral Palsy During a Lower Extremity Task. Neurorehabilit. Neural Repair.

[131] Zhao M., Gersch T.M., Schnitzer B.S., Dosher B.A., Kowler E. (2012). Eye Movements and Attention: The Role of Pre-Saccadic Shifts of Attention in Perception, Memory and the Control of Saccades. Vision. Res..

